# Delirium in older hospitalized patients—signs and actions: a retrospective patient record review

**DOI:** 10.1186/s12877-018-0731-5

**Published:** 2018-02-06

**Authors:** Yvonne A. Johansson, Ingrid Bergh, Iréne Ericsson, Elisabeth Kenne Sarenmalm

**Affiliations:** 1 Skaraborg Hospital, Skövde, Sweden; 2 University of Skövde, Skövde, Sweden; 3 Jönköping University, Jönköping, Sweden

**Keywords:** Signs of delirium, Neurocognitive disorders, Older hospitalized patients, Person-centered care, Patient safety, Patient participation, Action by healthcare professionals, Qualitative content analysis

## Abstract

**Background:**

Delirium is common in older hospitalized patients, and is associated with negative consequences for the patients, next of kin, healthcare professionals and healthcare costs. It is important to understand its clinical features, as almost 40% of all cases in hospitals may be preventable. Yet, delirium in hospitalized patients is often unrecognized and untreated. Few studies describe thoroughly how delirium manifests itself in older hospitalized patients and what actions healthcare professionals take in relation to these signs. Therefore, the aim of this study was to describe signs of delirium in older hospitalized patients and action taken by healthcare professionals, as reported in patient records.

**Methods:**

Patient records from patients aged ≥65 (*n* = 286) were retrospectively reviewed for signs of delirium, which was found in 78 patient records (27%). Additionally, these records were reviewed for action taken by healthcare professionals in relation to the patients’ signs of delirium. The identified text was analyzed with qualitative content analysis in two steps.

**Results:**

Healthcare professionals responded only in part to older hospitalized patients’ needs of care in relation to their signs of delirium. The patients displayed various signs of delirium that led to a reduced ability to participate in their own care and to keep themselves free from harm. Healthcare professionals met these signs with a variation of actions and the care was adapted, deficient and beyond the usual care. A systematic and holistic perspective in the care of older hospitalized patients with signs of delirium was missing.

**Conclusion:**

Improved knowledge about delirium in hospitals is needed in order to reduce human suffering, healthcare utilization and costs. It is important to enable older hospitalized patients with signs of delirium to participate in their own care and to protect them from harm. Delirium has to be seen as a preventable adverse event in all hospitals units. To improve the prevention and management of older hospitalized patients with signs of delirium, person-centered care and patient safety may be important issues.

## Background

Delirium is the most common complication in older hospitalized patients [1, 2] and is associated with negative hospital outcomes [3–7], including an increased risk of falling, prolonged hospital stays, cognitive and functional decline, mortality, increased healthcare utilization and increased costs [8, 9]. Still, delirium is often unrecognized [10] or poorly understood and managed in hospitals [11].

Delirium mainly occurs in older people exposed to stress, including acute illness and hospitalization [12]. The frequency among older hospitalized patients is up to 30% with higher rates among patients with frailty, hip fractures, advanced cancer [8] and at the end of life [2, 6, 12].

Delirium can be defined as acute brain failure occurring in persons with diminished reserve capacity [13], e.g., brain aging with greater sensitivity [9]. It is commonly due to underlying causes and is, in general, reversible when the underlying etiological factors are treated [2]. The etiology is complex and multifactorial [8, 12]. Predisposing and precipitating factors interact and patients with many predisposing factors may develop delirium easily [10]. The most important risk factor for delirium is cognitive impairment [2], and at least two third of all cases of delirium occur in patients with a preexisting neurocognitive disorder (NCD), e.g., Alzheimers disease [10].

It is important to understand the clinical features of delirium, as it is a clinical bedside diagnosis [9, 11]. Signs of delirium are disturbed attention, awareness and cognition that develop over a short period of time and fluctuate in severity. Hyperactive delirium is easiest to recognize with increased psychomotor activity and often mood fluctuations, agitation, refusal to co-operate, disruptive behavior, disturbance in the sleep-wake cycle and hallucinations. Hypoactive delirium, which is more common among the oldest [2] and in palliative care [14], is characterized by reduced psychomotor activity, sluggishness and lethargy. Rapid fluctuations between hyper- and hypoactive psychomotor activity, as well as a normal level of psychomotor activity with disturbed attention and awareness, indicate mixed delirium [2].

International guidelines and research describe how to prevent and treat delirium [9, 15–18]. Despite that, and the fact that almost 40% of all cases of delirium in hospitalized patients may be preventable [18], it may be unrecognized in up to two-thirds of cases [12, 16, 19], not thought to be clinically relevant or mistaken for NCD, but also depression [11, 12] or fatigue [14]. Ageist attitudes with an expectation of ‘disorientation’ may contribute to missed diagnoses [11, 20, 21], as well as ignorance and lack of interest in geriatric care issues [21].

Although the signs of delirium are clearly described in the Diagnostic and Statistical Manual of Mental Disorders (DSM-5) [2], there is poor recognition and management by healthcare professionals of hospitalized patients with these signs [10, 11]. There are few studies that thoroughly describe which signs of delirium healthcare professionals report in the patient records and what action they take in relation to these signs. Therefore, this study aims to describe signs of delirium in older hospitalized patients and action taken by healthcare professionals, as reported in patient records.

## Methods

### Design and setting

The study design was descriptive with a qualitative approach. A retrospective patient record review of somatic inpatients in a county hospital with more than 700 beds in western Sweden was conducted. The county has approximately 260,000 inhabitants.

### Sample and data collection

The first author (YJ) performed all the patient record reviews. The inclusion criteria in this study were patients aged ≥65, with signs of delirium reported in the patient records at admission or/and during the entire hospital stay.

In the years 2011–2012, 480 patient records were reviewed in the hospital to identify adverse events with the Global Trigger Tool method [22, 23]. From approximately 2500 somatic inpatient records monthly, 20 patient records per month were randomly selected using the Minitab®17 statistics software. Patients aged ≥65 were selected from these 480 records, which resulted in 286 inpatients whose records were reviewed according to signs of delirium and action taken by healthcare professionals in relation to these signs.

#### Sociodemographic and medical data

A patient record review protocol was developed with variables such as gender, age, residence, type of hospital admission, length of hospital stay, medical specialty at discharge, death during hospitalization, main diagnosis [24], comorbidity and a dementia diagnosis.

#### Signs of delirium

Descriptions of signs of both hyperactive, hypoactive and mixed delirium in patient records were reviewed using a protocol based on the diagnostic features of delirium in the DSM-5 [2], e.g., a disturbance in attention and awareness. The timing of the signs was also reviewed.

#### Action taken by healthcare professionals

The review included action taken by healthcare professionals in relation to the patients’ signs of delirium. Healthcare professionals who had reported in the patient records were physicians, registered nurses (RN), occupational therapists (OT), physiotherapists (PT) and speech therapists.

### Analysis

Two qualitative content analyses according to Elo and Kyngäs [25] were performed, first an inductive analysis of patient signs of delirium, and then a deductive analysis of action taken by healthcare professionals in relation to these signs. In both analyses, the data were organized in a stepwise categorization process. First, text transferred from the review protocols was divided into two documents: signs and actions. In each analysis, the text was read through several times to get a sense of wholeness of the content. The co-authors also read the text independently. Throughout the analyses, each step was discussed with the co-authors until consensus was reached.

The inductive analysis of patient signs of delirium included open coding, creation of subcategories and generic categories, and abstraction. Content with similarities were marked in the text as initial codes that were merged into subcategories based on similarities and dissimilarities of content. The generic categories were formed of subcategories in the same way. Codes, subcategories and generic categories were continuously moved back and forth, and checked against the original text. Finally, the generic categories were abstracted into a main category.

The deductive analysis of actions was then performed and followed one of two deductive ways described by Elo and Kyngäs [25]. First, the generic categories and subcategories from the analysis of patient signs of delirium were used as a coding scheme and the content of the actions was sorted in that scheme. Subcategories, generic categories and a main category were then formed out of the content of the actions, in the same way as in the analysis of the patient signs of delirium.

## Results

### Sample characteristics

Of the 286 inpatients aged ≥65 years, 78 patients (27%) had reports of signs of delirium in their records, and they constituted the sample in this study. Sample characteristics are presented in Table 1.

**Table 1:** Sample characteristics of older hospitalized patients with signs of delirium reported in their patient records

Variables	Women	Men	Total
Gender, *n* (%)	48 (62%)	30 (38%)	78
Age, mean (min-max)	83 (65–98)	80 (67–99)	82 (65–99)
Acute admission, *n*	48	30	78
Length of hospital stay, days			
Mean (min-max)	9.1 (1–35)	12.2 (1–35)	10.3 (1–35)
Medical specialty, at discharge			
Surgical wards, *n* (%)	16 (33%)	8 (27%)	24 (31%)
Medical wards, *n* (%)	32 (67%)	22 (73%)	54 (69%)
Residence, *n* (%)			
Living alone	21 (44%)	4 (13%)	25 (32%)
Living with	8 (17%)	16 (53%)	24 (31%)
Institutionalized care^a^	19 (39%)	10 (33%)	29 (37%)
Dementia, diagnosed, *n* (%)	12 (25%)	7 (23%)	19 (24%)
Dementia, suspected, *n* (%)^b^	5 (10%)	1 (3%)	6 (8%)
Died during hospital stay, *n* (%)	5 (10%)	4 (13%)	9 (11.5%)

^a^Including short-term care, dementia care and respite care. ^b^ Investigation underway/planned, and memory problems

### Hospitalization characteristics

The reasons for hospital admission for the 78 patients were, for some patients, living in institutionalized care, repeated falls, contusions, lethargy, or carbon dioxide retention caused by sleep medication, analgesics or sedatives. Need of care planning led to hospitalization for some patients with unsustainable home situations, where the patients had deteriorated with disorientation, anxiety, poor general condition, aggression, personality changes, memory problems, sleeping difficulties and/or falls. One patient had been lying on the floor for several days without being found. Another patient had left home at night and had trouble finding the way back. One patient took care of his sick wife at home, which was considered unsustainable. The most common main diagnosis groups were vascular diseases (*n* = 19) and injuries (*n* = 14). Of the patients who died in hospital, all but two lived in institutionalized care before hospitalization.

### Signs and actions

Firstly, the inductive content analysis of older patients’ signs of delirium is reported in a table (Table 2) and in the text, with the generic categories as headings. The deductive analysis of action taken by healthcare professionals is then reported in a table (Table 3) and in the text, with the generic categories as headings. Under each action heading, the generic categories of patient signs of delirium are used as subheads and the subcategories of action taken are described under those subheads.

**Table 2:** Signs of delirium in older hospitalized patients as reported in patient records

Subcategories	Generic categories	Main category
Problems remembering	Difficulty describing their situation	Reduced ability to participate in their own care and to keep themselves free from harm
Conflicting and doubtful information		
Problems verbalizing symptoms and needs		
Problems managing personal care and mobilization	Difficulty taking care of themselves	
Lack of initiative		
Risked falling and injuring themselves		
Non-compliance		
Disoriented	Difficulty interpreting reality	
Hallucinations		
Altered day-night perception		
Impaired awareness		
Problems understanding information and instructions		
Anxious, sad and insecure	Difficulty handling their emotions	
Agitated and aggressive

**Table 3:** Action taken as reported by healthcare professionals when older hospitalized patients had signs of delirium

Subcategories	Generic categories	Main category
Considered the signs of delirium	Adapted care	Variation in actions
Communicated in several ways		
Need for care after hospitalization		
Support and physical assistance		
Adjusted the medical and nursing care		
Subjective interpretations	Deficient care	
Relied on patients’ abilities		
Disregarded signs of delirium		
Extra measures and extra care	Beyond usual care	
Care was withdrawn		

### Signs of delirium reported in patient records

The inductive content analysis of older hospitalized patients’ signs of delirium resulted in a main category: Reduced ability to participate in their own care and to keep themselves free from harm. The main category consisted of four interrelated generic categories: (1) Difficulty describing their situation; (2) Difficulty taking care of themselves; (3) Difficulty interpreting reality, and (4) Difficulty handling their emotions. The generic categories were generated from 14 subcategories (Table 2).

### Main category: Reduced ability to participate in their own care and to keep themselves free from harm

Reported signs of delirium in older hospitalized patients revealed that the patients had various signs of delirium, with difficulty describing their situation, taking care of themselves, interpreting reality and handling their emotions. Together, these signs resulted in a reduced ability to participate in their own care and to keep themselves free from harm.

#### Generic category: Difficulty describing their situation

The patients had problems remembering their medical history, their social situation, why they were in hospital or how long they had been there *“Has a scar on the abdomen but cannot remember what the surgery was for”* (Physician). They could not describe how they lived, e.g., the layout of their houses or how they had managed at home.

Conflicting and doubtful information was given by the patients. Their information was not consistent with information from their next of kin, from staff members or in their records *“Confused on arrival* (at the hospital)*. Says he slept well last night and took a morning walk; however, found on the bathroom floor in the morning”* (Physician). Different information was given to different healthcare professionals and sometimes the patients changed their narrative during a conversation. Despite signs, e.g., of dyspnea the patients sometimes denied symptoms.

The patients had problems verbalizing symptoms and needs. It was described that the patients responded with latency, had trouble finding words, were messy and irrelevant or asked the same question repeatedly. Some patients did not talk at all or did not want to answer. Others muttered incoherently, sometimes to themselves *“The patient replies happily when spoken to, but is totally incomprehensible”* (Physician). The patients had signs that were interpreted as pain, e.g., agitation, disorientation, crying and grimacing *“Somewhat anxious in the evening, talks about chest pain and then about tubes; infusion tubes and oxygen tubes”* (RN). The patients screamed and held different body parts or were completely immobile after their analgesics were withdrawn. Still, some of them denied being in pain and although they asked for special analgesics they could not specify the pain location.

#### Generic category: Difficulty taking care of themselves

The patients had problems managing personal care and mobilization. Major needs of help emerged but many refused help *“Was offered a shower but refused outright”* (RN). The patients did not understand how things should be used, forgot what to do or continued with an activity until someone stopped them. Dirty underwear or diapers were not changed and some patients smeared themselves with feces *“Believes that she has performed her personal hygiene routines in the morning but has not changed her clothes, which are dirty”* (OT). The patients had problems handling new aids and remembering and performing exercises, both with and without assistance*.*

Lack of initiative was reported when patients were described as passive, apathetic, slightly absent and difficult to mobilize. The patients would rather stay in bed and did not initiate any activities *“Seen as somewhat apathetic, lies on his/her bed most of the day with a hat on”* (RN).

The patients risked falling and injuring themselves but were described as being unaware of this. Sometimes falls were the reason for hospitalization but most patients did not remember falling *“X-ray shows an old collum fracture; unclear how the fracture happened”* (Physician). Some patients fell repeatedly in the hospital, especially at night, which occasionally resulted in injuries *“Fell at night when he was walking around with his walker; pain in hip and leg shortened”* (RN). The patients’ gait was unsteady and they supported themselves on furniture and walls. Disorientation, agitation and aggressiveness often occurred simultaneously with falls *“Fell again last night… The patient climbed over the bed rails and fell down on the floor… Is very disoriented and confused”* (RN).

There was presence of non-compliance*;* the patients refused care, examination and treatment, and did not participate or cooperate *“The patient started crying… he does not want to be examined and does not cooperate”* (Physician). The patients sometimes refused taking their medication, although it was started in cooperation with them when they had requested treatment. Some patients spat out their pills. It was described that the patients struggled desperately against attempts by healthcare professionals to move them in the bed; they refused sampling, infusions and bladder catheters, as well as eating, washing, taking deep breaths, or using dentures or hearing aids. At mobilization, patients were annoyed and wanted to be left in peace *“Will try walking with a walker… but the patient is very determined not to try it today”* (PT). One patient smoked in bed in the hospital.

#### Generic category: Difficulty interpreting reality

The patients were disoriented about events, time, places and people, especially in evenings and at night. They could not identify being in the hospital wards *“In a cognitive test, the patient states that she is in a library in a town* (names another town)*”* (OT). The patients were described as being disoriented during all or a part of the hospital stay, e.g., postoperatively. They often used the buzzer to call the healthcare professionals or yelled loudly.

Sometimes, the patients had hallucinations, mostly at night, together with anxiety and screaming. The patients said they mixed up dreams and reality and saw people and dead friends in their rooms *“Was anxious at night, hallucinated and had delusions; thinks she will be abducted”* (Physician).

There was also altered day-night perception. The patients had difficulty getting restful in the evening; they were worried and put on their clothes to go home. They slept a few hours or not at all, instead they slept a lot during the day *“The patient does not know whether it is day or night”* (RN).

The patients had impaired awareness of their abilities. Cognitive testing revealed deficiency of attention, judgment and safety for some patients who, nevertheless, wanted to handle their medication after discharge. Some of them were hospitalized because they had forgotten to take their medication or had taken the whole daily dose at the same time *“Sometimes forgets to take his/her medication at home”* (Physician).

The patients had problems understanding information and instructions. They had problems learning new things and describing information they had been given, e.g., why new treatments were needed. Instructions were not followed *“Difficult to examine field of vision, as the patient is unable to focus but looks at his/her hands the whole time”* (Physician). At auscultation of the heart/lungs they were muttering. They chewed depot pills that should be swallowed whole and sucked instead of blew in the Bubble PEP postoperatively. The patients supported themselves on operated-on fractures that must not be exposed to strain, despite reprimands and healthcare professionals being present *“Supports him/herself excessively on the fractured arm when using the walker and gets more pain”* (PT). Some patient had to be committed to bed rest to avoid putting strain on fractures. One patient who had a plaster cast on an amputation stump removed the plaster repeatedly at night, even though this was described as being impossible *“The patient has removed the plaster cast again at night and protests when attempts are made to put it back on*” (RN).

#### Generic category: Difficulty handling their emotions

The patients were described as anxious, sad and insecure, mostly during nighttime *“Calls the staff and is worried about not being able to reach the buzzer that she has just used at night”* (RN). The patients had concerns about relatives, examinations, treatments, change of hospital wards or hospital discharge and were described as having great contact needs *“The patient wakes up crying and wants her husband to come”* (RN). The patients were described as being depressed and dejected with reduced well-being. They were tearful and felt that no one cared about them *“Says that she does not want to live; sees no point in being alive”* (RN).

During examinations and in caring situations, several patients became agitated and aggressive. It was documented that the patients behaved in a threatening and unpleasant manner and were acting out. They also shouted, screamed, punched and kicked *“Wanted a pill at night but did not take what was offered, but gets aggressive and tells staff they are holding the glass the wrong way. Gets very angry and abusive and waves his/her hand against the staff”* (RN).

During episodes of anxiety and agitation, the patients were ‘picking’ and pulling the most, often at night*.* They removed dressings, infusions, catheters and monitoring devices *“Removed the telemetry monitoring device herself and collapsed suddenly”* (Physician). After pulling out the urinary catheter, with subsequent complications, the patient became aggressive and would not cooperate *“Urinary catheter insertion attempted during the night, but the patient is very angry, screams and shouts and lashes out at the staff”* (RN).

### Action taken in relation to patients’ signs of delirium

The deductive content analysis of action taken by healthcare professionals related to patients’ signs of delirium resulted in the main category; Variation in actions. Three generic categories formed the main category: (1) Adapted care; (2) Deficient care; (3) Beyond usual care. The generic categories were generated from ten subcategories (Table 3).

### Main category: Variation in actions

Reported action taken by healthcare professionals in relation to older hospitalized patients’ signs of delirium revealed that there was a variation in actions and the care was both adapted, deficient and beyond usual care.

### Generic category: Adapted care

#### When patients had difficulty describing their situation

Sometimes, healthcare professionals *considered the signs of delirium* and cognitive tests were carried out. The professionals *communicated in several ways* with the patients to understand what the patients meant. It was described as essential that the patients were given enough time to express themselves.

#### When patients had difficulty taking care of themselves

Healthcare professionals c*ommunicated in several ways* with the patients, trying to get them to participate in their own care, and gestures and objects could be used. They described that it was difficult to know whether the patients could not or would not participate. The patients’ *need for care after hospitalization* was identified. Assessments of Activities of Daily Living (ADL) and care planning were made at the hospital or in the patient’s home after discharge. Healthcare professionals gave *support and physical assistance* with ADL and exercises in training programs. They *adjusted the medical and nursing care.* Extra energy could be given and energy and fluid intake measured. In some cases, fall risk assessments and fall prevention were carried out and bed rails used.

#### When patients had difficulty interpreting reality

Healthcare professionals sometimes *considered the signs of delirium.* Medication might be considered to cause or contribute to adverse effects like disorientation and aggression, and long-acting medication, for instance, was discontinued. Cognitive tests were sometimes performed and disorientation followed up after discharge. Healthcare professionals c*ommunicated in several ways* with the patients to try to get them to absorb information. It was described as essential to speak clearly and slowly and for the information to be brief and repeated, but also that it was hard to know how much the patients understood. Written information was sometimes considered. The patient’s next of kin was informed about the patient’s condition and exercise programs, and were sometimes given information leaflets. Occasionally, healthcare professionals *adjusted the medical and nursing care.* Medication administration was modified and tablets crushed. To avoid disorientation, healthcare professionals avoided moving a patient from one hospital to another, even if the patient’s physical condition allowed it. Disoriented patients could be given hot beverage in the evening to calm them down.

#### When patients had difficulty handling their emotions

Healthcare professionals *communicated in different ways* to calm the patients. Being close, taking time to communicate and being careful and gentle could be more effective than sedatives and/or analgesics and the patients could talk about their symptoms, anxiety and thoughts. Healthcare professionals also a*djusted the medical and nursing care.* A quiet and safe environment was described as important. Sometimes, the patients needed to be left alone to calm down. One husband was called at 06:00 AM to calm down his wife who had not been helped by other actions.

### Generic category: Deficient care

#### When patients had difficulty describing their situation

*Subjective interpretations* of patient sign were made. Pain assessment tools were used a few times and then postoperatively. Tools adapted to patients with cognitive impairment were not used at all. Pain was often described as “seems to/appears to/may have pain”. It was reported as difficult to assess the level of pain postoperatively, after fall events, and to distinguish pain from anxiety. Sedatives and/or analgesics were given with various effects. Opioid antagonist was required postoperatively due to repeated pain medication when anxiety had been interpreted as pain. Healthcare professionals *relied on patients’ abilities* and prescribed medication, for instance, antibiotics, when patients who had difficulty describing their situation said they tolerated all food and all medication.

#### When patients had difficulty taking care of themselves

Healthcare professionals *disregarded signs of delirium.* Weight or/and Body Mass Index (BMI) were not always measured although it was described that the patient’s main problem was food intake. Bed rails were sometimes erected again when patients climbed over them and fell to the floor, which resulted in new falls. Healthcare professionals *relied on patients’ abilities.* They encouraged patients with signs of delirium to take initiatives and responsibilities, e.g., to eat, drink and mobilize, and also recommended them to perform movements and breathing exercises periodically.

#### When patients had difficulty interpreting reality

Healthcare professionals *disregarded signs of delirium* and *s*ometimes the patients were given a lot of information simultaneously. It was frequently described that the patient was disoriented but no action was taken. Necessary surgery for patients with dementia diseases and/or disorientation was postponed, due to other than medical reasons, sometimes for several days, with infusions and sedatives instead of food and beverage. Because of a lack of hospital beds, patients with disorientation could be moved from a room to the corridor where their disorientation increased. Mostly *subjective interpretations* regarding the patients’ cognitive ability were expressed as “seems as, is experienced as, appears”, “a little-partly-very” “disoriented-confused-bemused”. The word delirium was only used by psychiatrists. Assessment tools for cognitive ability were rarely used and for delirium not at all. Healthcare professionals also *relied on patients’ abilities,* as disoriented patients were considered to have understood information about operations, for instance, amputation.

#### When patients had difficulty handling their emotions

Healthcare professionals *disregarded signs of delirium* when worried patients were placed in single rooms with a closed door and the patients yelled loudly. *Subjective interpretations* were performed of patients’ signs of emotional instability.

### Generic category: Beyond usual care

#### When patients had difficulty describing their situation

*Extra measures and extra care* were performed. Information was retrieved from different sources, such as patient records, next of kin or accompanying staff, sometimes by phone. Medication lists, if available, were used to understand what diseases the patients had. Extra samplings and examinations could be needed, for instance, blood samples and X-rays.

#### When patients had difficulty taking care of themselves

*Extra measures and extra care* were performed. Security guards who worked in the hospital helped to lift patients who had fallen, and patients with fractures due to falls in the hospital underwent surgery. In case of non-compliance and refusal, help was sometimes sought in other units, for instance, from an anesthetist in connection with X-ray examinations. Infusions and total parenteral nutrition might be given when the patients refused to eat and drink. When strain was put on operated-on fractures, despite instructions not to do so, renewed X-rays and, e.g., circular plaster casts and wheelchairs were prescribed. Sometimes the *care was withdrawn*, especially if it was considered impossible for the patients to cooperate when required or when the patient did not want to participate, e.g., in pressure relief in the case of pressure ulcers.

#### When patients had difficulty interpreting reality

*Extra measures and extra care* were performed. During evenings and nights registered nurses contacted the physician on call for prescription of medication to treat hallucinations and disorientation.

#### When patients had difficulty handling their emotions

*Extra measures and extra care* were performed. Psychiatrists were consulted in cases of severe agitation and registered nurses contacted the physician on call in the evening/night for prescription of sedatives and/or analgesics, or for administration changes. They also asked the day shift to consider sedative prescriptions. Several healthcare professionals were required to give injections to agitated patients. When aggressive and agitated patients pulled off dressings and tubes, new ones were applied or inserted and additional samples could be taken. Sometimes the patients were supervised at frequent intervals but all-time surveillance was rare. Occasionally the *care was withdrawn*, and it was sometimes decided to withdraw infusions, bladder catheters and monitoring devices before the patients did it themselves.

In summary, the results revealed that the variation in actions taken by healthcare professionals in relation to older hospitalized patients’ signs of delirium responded only in part to the patients’ need of care in relation to these signs. The care was not systematic and consistent. To each sign of delirium both adapted care, deficient care and care beyond usual care could be given.

## Discussion

The main finding in this study is that variation in action by healthcare professionals responded only in part to older hospitalized patients’ need of care related to their signs of delirium.

Older hospitalized patients with signs of delirium, as reported in patient records, had a reduced ability to participate in their own care and to keep themselves free from harm, and healthcare professionals’ actions varied, as the care was adapted, deficient and beyond usual care. Even if healthcare professionals seemed to identify and meet the patients’ physical care needs, they often failed to meet other needs and provided deficient care; for instance, causes of disorientation were not considered and many signs of delirium seemed to have been left without subsequent action.

Signs of delirium were described to occur more frequently during evenings and nights. It is known that delirium often occurs or increases at that time [2, 26, 27], when staffing levels are the lowest. This probably affects all patients in hospital wards. The findings indicate that older patients with signs of delirium generated a high workload for healthcare professionals, especially when the patient exhibits symptoms like refusals and aggressiveness, which is in line with previous research [28]. Study results also show that there seems to be more frequent difficulties in orthopedic wards, or problems that occur only in such wards. Many orthopedic patients needed but could not manage walking aids and had difficulty following postoperative regimes, which led to increased pain and jeopardized treatment outcomes.

Patients with signs of delirium may have severe difficulties verbalizing their symptom experience and care needs. Therefore, early identification of these patients’ signs and symptoms, evaluated by using reliable assessment tools, is utterly important. Nevertheless, study findings indicate that professionals mainly interpreted patient signs subjectively, and cognitive ability, delirium and pain were described with vague expressions. Many patients had signs that were interpreted as pain but it was described as difficult to know whether the signs reflected pain or not. It is possible that the patients received inadequate pain relief, which could have affected the signs of delirium negatively, as it is known that pain may cause and maintain delirium [26]. Other terms than delirium were used and it is unclear whether these expressions meant the same thing to all healthcare professionals or if similar assessments had been carried out. Previous research indicates that healthcare professionals may have a cultural understanding of the concept of delirium and are unfamiliar with using the word, as it is commonly associated with alcohol abuse [29].

It is important to ensure the safety of these patients [18] as, according to the findings in this study, they unintentionally cause themselves harm, for instance, through fall-related injuries and the negative implications of their refusals. Healthcare professionals seemed to be unaware of that, as, according to patient records, systematic prevention and treatment of delirium were mostly missing and the patients were not kept safe and protected. Additionally, a systematic evaluation of underlying causes to delirium was rare, even though delirium is commonly due to underlying causes [2]. There is a risk that several patients in the study had treatable conditions that were not treated. It is essential to emphasize the seriousness of delirium. As almost 40% of all cases of delirium are considered preventable [18], delirium and subsequent harm have to be seen as preventable adverse events [30]. The findings also indicate that delirium is an adverse event in all hospital units and not just in Intensive Care Units, as considered in the revised patient safety tool recommended by The Swedish Association of Local Authorities and Regions in Sweden [31]. To improve the prevention and management of delirium, this condition should be included in that tool as a preventable adverse event in all hospital units.

There is a requirement in the legalization that all patients should be given the opportunity to participate in their own care [32], and it is important to enable these patients to participate, as their ability to do so is reduced.

The signs of delirium in this study were in accordance with previous knowledge [2], but the results were described at a detailed level, which may be more recognizable for healthcare professionals. Moreover, patients’ inability to participate in their own care and their risk of harming themselves became very clear. Additionally, the findings not only confirmed that older patients with signs of delirium are poorly managed in hospitals [11], but also identified the failures. It seems that care beyond usual care was required when healthcare professionals were one step behind to meet the patients’ needs in relation to their signs of delirium and the patients’ signs of delirium were thereby exacerbated. This is in line with previous research, which has also shown a lack of consensus in the care [33], like the findings in this study. If adapted care had been given, which is in line with available evidence [9, 15–18], patients’ suffering, harm and other negative consequences of delirium had probably decreased. The amount of care beyond usual care and, thereby, the workload of the healthcare professionals, had very likely decreased, as the incidence, duration and severity of the delirium would have been reduced. Adapted care is also in line with person-centered care where every patient is seen as a person with individual needs and where every patient’s abilities and resources are fundamental [34]. It is unclear whether it was merely lack of knowledge that affected the action taken, or negligence, as geriatric issues can be perceived as unchallenging in hospitals [21], although advanced care is required for older hospitalized patients, just as for younger patients in early life [35]. With improved knowledge about delirium in hospitals, deficient care might not exist.

### Limitations and strengths

Throughout the study, the intention has been to establish trustworthiness, which includes the criteria of credibility, dependability, confirmability and transferability [36–38]. A limitation of the study is the retrospective identification of patients’ signs of delirium and healthcare professionals’ actions, which is strongly dependent on paying attention to patients’ signs and reliable recording in the patient records. The descriptions of cognitive ability at hospital admission and discharge may sometimes be sparse [39, 40], but in this study, the amount of descriptions of signs of delirium during hospital stays was extensive. This suggests that signs of especially hyperactive delirium are very noticeable. However, the reporting of action taken was sparse and often described with only a few words. Commonly, no reported action followed descriptions of the sign of disorientation, but there might have been actions as well as signs of delirium that were not reported, which may affect the credibility of the study. Even if the data about actions consisted of short phrases, saturation was established in both content analyses, which is a prerequisite for trustworthiness. The prevalence of signs of delirium (27%) was also in line with previous knowledge [2].

The first author conducted all the patient record reviews. To strengthen confirmability, a structured review protocol was used to ensure that the same information was obtained from all patient records. Discussions with the co-authors until consensus throughout the study strengthened the credibility and confirmability, as did the quotations from as many patients as possible. Moreover, the study sample size was large, and patients from different medical specialties with various diagnoses were represented in the sample. Furthermore, all documentation in the patient records by all categories of healthcare professional was reviewed. Together with as thorough descriptions as possible throughout the research process this may facilitate the transferability of the findings to similar contexts. Despite the limitations of the study, the findings provide an insight into what signs of delirium older hospitalized patients may have and what actions healthcare professionals may take in relation to these signs.

## Conclusion

A systematic and holistic perspective of the care of older hospitalized patients with signs of delirium was missing and the patients seemed to be exposed and vulnerable. Adapted care was a good example of action taken, but generally, healthcare professionals did not respond to these patients’ needs of care in relation to their signs of delirium. Many actions were reactive instead of anticipative and preventive, and there was even deficient care. As the population and patients in hospitals are getting older [41], it is important to improve the knowledge about delirium and its prevention and management among healthcare professionals. Moreover, to enable these patients to participate in their own care and protect them from harm, person-centered care and patient safety are important issues. Validated assessment tools for different signs also have to be used. Additionally, the term “delirium” could help emphasize that disorientation and other signs of delirium are not normal signs in older hospitalized patients. Delirium has to be seen as a preventable adverse event in all hospitals units, and the incidence may be used as a quality indicator like, for instance, in the Netherlands [17]. In Sweden, there are so far no national guidelines about the prevention and management of delirium in hospitals, as there are, for example, in the UK, the US and Australia [9, 15, 16]. Further research and interventions are needed in relation to the prevention and management of delirium in hospitals.

## Abbreviations

Bubble-PEP: Bubble Positive Expiratory Pressure. Breathing techniques with a positive expiratory pressure (PEP) are used to increase airway pressure and improve pulmonary function. The technique involves blowing bubbles through water

## References

[1] Rice K, Bennett M, Gomez M, Theall K, Knight M, Foreman M (2011). Nurses’ recognition of delirium in the hospitalized older adult. Clin Nurse Spec.

[2] American Psychiatric Association. Diagnostic and statistical manual of mental disorders, DSM-5. Fifth edition. 5th. Washington DC: American Psychiatric Publishing. 2013.

[3] Andersson EA, Hallberg I-LR, Norberg A, Edberg A-K (2002). The meaning of acute confusional state from the perspective of elderly patients. Int J Geriatr Psychiatry.

[4] Stenwall E (2009). Ett ögonblick i sänder – mötet vid akut förvirringstillstånd, äldre patienters, närståendes och professionella vårdares perspektiv. (Diss.).

[5] Witlox J, Eurelings LS, de Jonghe JF, Kalisvaart KJ, Eikelenboom P, Van Gool WA (2010). Delirium in elderly patients and the risk of postdischarge mortality, institutionalization, and dementia: a meta-analysis. JAMA.

[6] Lawlor PG, Bush SH (2015). Delirium in patients with cancer: assessment, impact, mechanisms and management. Nat Rev Clin Oncol.

[7] Yue P, Wang P, Liu C, Wu Y (2015). A qualitative study on experience of nurses caring for patients with delirium in ICUs in China: barriers, burdens and decision making dilemmas. International Journal of Nursing Sciences.

[8] Siddiqi N, Holt R, Britton AM, Holmes J. Interventions for preventing delirium in hospitalised patients. Cochrane Database of Syst Rev. 2007; Issue 2. Art. No.: CD005563. https://www.scribd.com/document/56840384/Interventions-for-Preventing-Delirium-in-Hospital-is-Ed-Patients. Accessed 22 Sept 2017. 10.1002/14651858.CD005563.pub2 17443600

[9] American Geriatrics Society. The American Geriatrics Society/National Institute on Aging beside-to-bench conference: research agenda on delirium in older adults. AGS/NIA delirium conference writing group, planning committee and faculty. JAGS. 2015a;63:843-852. 10.1111/jgs.13406 PMC5407494 25834932

[10] Inouye SK (2006). Delirium in Older Persons. N Engl J Med.

[11] Saxena S, Lawley D (2009). Delirium in the elderly: a clinical review. Postgrad Med J.

[12] Fong TG, Tulebaev SR, Inouye SK (2009). Delirium in elderly adults: diagnosis, prevention and treatment. Aging brain center, Institute for Aging Research, Hebrew senior life, Boston, MA, USA. Nat Rev Neurol.

[13] Inouye SK, Westendorp RG, Saczynski JS (2014). Delirium in elderly people. Lancet.

[14] Spiller JA, Kern JC (2006). Hypoactive delirium: assessing the extent of the problem for inpatient specialist palliative care. Palliat Med.

[15] National Institute for Health and Care Excellence (NICE). Delirium: prevention, diagnosis and Management. Clinical guideline. nice.org.uk/guidance/cg103. Manchester. 2010. 31846262

[16] Australian Commission on Safety and Quality in Healthcare. Evidence for the safety and quality issues associated with the care of patients with cognitive impairment in acute care settings: a rapid review, ACSQHC, Sydney. Authors: Travers C, Gray L, Martin-Khan M, Hubbard R. Centre for Research in Geriatric Medicine. The University of Queensland. UniQuest Project No: C01397. 2013. https://www.safetyandquality.gov.au/wp-content/uploads/2013/10/Rapid-Review_Evidence-for-the-safety-and-care-of-patients-with-cognitive-impairment-in-acute-care-settings.pdf. Accessed 22 Sept 2017.

[17] Strijbos MJ, Steunenberg B, van der Mast RC, Inouye SK, Schuurmans MJ. Design and methods of the Hospital Elder Life Program (HELP), a multicomponent targeted intervention to prevent delirium in hospitalized older patients: efficacy and cost-effectiveness in Dutch healthcare. BMC Geriatr. 2013;13:78. https://www.biomedcentral.com/1471-2318/13/78. Accessed 22 Sept 2017. 10.1186/1471-2318-13-78 PMC3724594 23879226

[18] American Geriatrics Society. American Geriatrics Society abstracted clinical practice guideline for postoperative delirium in older adults. The American Geriatrics Society expert panel on postoperative delirium in older adults. JAGS. 2015b;63:142-150. 10.1111/jgs.13281 PMC5901697 25495432

[19] Inouye SK, Schlesinger MJ, Lydon TJ (1999). Delirium: a symptom of how hospital care is failing older persons and a window to improve quality of hospital care. Am J Med.

[20] Royal College of Psychiatrists. Who Cares Wins. Improving the outcome for older people admitted to the general hospital: Guidelines for the development of Liaison Mental Health Services for older people. Royal College of Psychiatrists. 2005. ISBN 0 85316 253 0. http://www.rcpsych.ac.uk/pdf/whocareswins.pdf. Accessed 22 Sept 2017.

[21] Teodorczuk A, Rynish E, Milisen K. Improving recognition of delirium in clinical practice: a call for action. BMC Geriatr. 2012;12:55. www.biomedcentral.com/1471-2318/12/55, 10.1186/1471-2318-12-55. Accessed 22 Sept 2017. 10.1186/1471-2318-12-55 PMC3463439 22974329

[22] Landstingens Ömsesidiga Försäkringsbolag, Landstinget i Jönköpings län, Landstinget i Kalmar län & Landstinget i Östergötland. Strukturerad journalgranskning för att identifiera och mäta förekomst av skador i vården enligt metoden Global Trigger Tool. Handbok för patientsäkerhetsarbete. Institute for Health-care Improvement Innovation series 2007. Svensk översättning och anpassning. 2008. ISBN:978–91–633-3370-5.

[23] Griffin FA, Resar RK. IHI global trigger tool for measuring adverse events (2th ed.). IHI innovation series white paper. Cambridge, MA: Institute for Healthcare Improvement. 2009.

[24] World Health Organization (WHO). International Statistical Classification of Diseases and Related Health Problems 10th Revision (ICD-10)-2015-WHO Version for; 2015. http://apps.who.int/classifications/icd10/. Accessed 22 Sept 2017.

[25] Elo S, Kyngäs H (2008). The qualitative content analysis process. J Adv Nurs.

[26] Robinson S, Vollmer C, Rich C (2008). Aging and delirium: too much or too little pain medication?. Pain Manag Nurs.

[27] Weinrebe W, Johannsdottir E, Karaman M, Füsgen I (2015). What does delirium cost?: an economic evaluation of hyperactive delirium. Gerontol Geriat.

[28] Leslie DL, Inouye SK (2011). The importance of delirium: economic and societal costs. J Am Geriatr Soc.

[29] Larsson C, Granberg Axell A, Ersson A (2007). Confusion assessment method for the intensive care unit (CAM-ICU): translation, retranslation and validation into Swedish intensive care settings. Acta Anaesthesiol Scand.

[30] Zywiel MG, Hurley RT, Perruccio AV, Hancock-Howard RL, Coyte PC, Rampersaud YR (2015). Health economic implications of Perioperative delirium in older patients after surgery for a fragility hip fracture. J Bone Joint Surg Am.

[31] The Swedish Association of Local Authorities and Regions, SALAR (Sveriges Kommuner och Landsting, SKL). Markörbaserad journalgranskning. För att identifiera och mäta skador i vården. Handbok. 2014. IBSN 978–91–7164-847-1.

[32] Patientlag (2014:821). Svensk författningssamling. Sveriges Riksdag. www.riksdagen.se/sv/dokument-lagar/dokument/svensk-forfattningssamling/patientlag-2014821_sfs-2014-821. Accessed 22 Sept 2017.

[33] Nilsson A, Rasmussen BH, Edvardsson D (2013). Falling behind: a substantive theory of care for older people with cognitive impairment in acute settings. J Clin Nurs.

[34] Ekman I, Swedberg K, Taft C, Lindseth A, Norberg A, Brink E, Carlsson J, Dahlin-Ivanoff S, Johansson IL, Kjellgren K, Lidén E, Öhlén J, Olsson LE, Rosén H, Rydmark M, Sunnerhagen KS. Person-centered care—Ready for prime time, Eur J Cardiovasc Nurs. 2011; 10.1016/j.ejcnurse.2011.06.008. 10.1016/j.ejcnurse.2011.06.008 21764386

[35] Olofsson B (2007). Old people with femoral neck fracture. Delirium, malnutrition and surgical methods – an intervention program. (Diss.).

[36] Lincoln SY, Guba EG. Naturalistic inquiry. Thousand Oaks, CA: Sage. 1985.

[37] Polit DF, Beck CT. Nursing research: principles and methods. Philadelphia, PA: Lippincott Williams & Wilkins. 2012.

[38] Elo S, Kääriäinen M, Kanste O, Pölkki T, Utriainen K, Kyngäs H. Qualitative Content Analysis: A Focus on Trustworthiness. SAGE Open, January–March 2014;1–10. 10.1177/2158244014522633.

[39] Söderqvist A (2007). Bedömning av kognitiv förmåga hos äldre patienter med höftfraktur. (Diss.).

[40] Dodson JA, Truong TT, Towle VR, Kerins G, Chaudhry SI. Cognitive impairment in older adults with heart failure: prevalence, documentation, and impact on outcomes. Am J Med 2013;126(2):120-6. 10.1016/j.amjmed.2012.05.029. 10.1016/j.amjmed.2012.05.029 PMC3553506 23331439

[41] The Swedish Association of Local Authorities and Regions, SALAR (Sveriges Kommuner och Landsting, SKL). Utvecklingen i svensk hälso- och sjukvård - struktur och arbetssätt för bättre resultat. Stockholm. 2009. ISBN: 978–91–7164-465-7.

[42] The World Medical Association Declaration of Helsinki (WMA). Ethical Principles for Medical Research Involving Human Subjects. 2004; Adopted by the 18th WMA General Assembly. Helsinki, Finland. 1964.

